# A TREM-1 Polymorphism A/T within the Exon 2 Is Associated with Pneumonia in Burn-Injured Patients

**DOI:** 10.1155/2013/431739

**Published:** 2013-02-12

**Authors:** Fernando A. Rivera-Chávez, Ryan M. Huebinger, Agnes Burris, Ming-Mei Liu, Joseph P. Minei, John L. Hunt, Brett D. Arnoldo, Robert C. Barber

**Affiliations:** Division of Burn/Trauma/Critical Care, Department of Surgery, University of Texas Southwestern Medical Center, 5323 Harry Hines Boulevard, Dallas, TX 75390-9160, USA

## Abstract

*Background*. The triggering receptor expressed in myeloid cells (TREM-1) is a key mediator in the activation of the local inflammatory response during lung infections. We aimed to evaluate the effect of a functionally relevant TREM-1 single nucleotide polymorphism within the exon 2 (A→T) on the development of pneumonia in burn patients. *Objective*. To determine whether a single nucleotide polymorphism (SNP) within the exon 2 (A→T) in the TREM-1 gene is associated with ventilator-associated pneumonia (VAP) in burn-injured patients. *Methods*. 540 patients with ≥10% total body surface area (TBSA) burn injuries or inhalation injury were prospectively enrolled. The influence of a polymorphism (A→T) in exon 2 of the TREM-1 gene was evaluated for association with increased risk of pneumonia by logistic regression analysis. *Measurements and Main Results*. 209 patients met criteria for VAP. Multivariate regression analysis showed that, after adjustment for potential confounders, we found that carriage of the TREM-1 T allele is associated with more than a 3-fold increased risk of VAP (OR 6.3, 95% CI 4–9). *Conclusions*. A TREM-1 single nucleotide polymorphism within the exon 2 (A→T) is associated with the development of pneumonia in burn patients.

## 1. Introduction

Ventilator-associated pneumonia (VAP) is a common complication in patients receiving mechanical ventilation in the burn intensive care unit (BICU) [1]. VAP results in substantial increase in morbidity and mortality [2–4]. Even though pneumonia is very common in the burn patient, the clinical and demographic factors that might predict who is at risk for developing VAP are nonspecific [5–9]. Additionally, the accuracy of standard clinical and laboratory methods of VAP diagnosis remains under constant scrutiny, and there is increasing debate concerning whether VAP is a preventable disease [10, 11].

The type of insult and clinical presentation of the patient do not predict which individuals will develop VAP and subsequent progression to sepsis, multi-system organ failure, or death [8, 9, 12–14]. There is a wide range of clinical responses to similar types and degrees of insult (i.e., not all patients with the same degree of burn and physiological similarities will go on to develop pneumonia). Similarly, different patients will often respond differently to the same treatment regimen. These differences may well reflect genetic differences amongst individuals [15–19]. Significant associations have been observed between specific SNPs and clinical outcome after burn and trauma [20–26].

The triggering receptor expressed on myeloid cells-1 (TREM-1) gene is located on chromosome 6 and encodes a 30-kDa glycoprotein member of the immunoglobulin (Ig) superfamily [27]. TREM-1 is a key mediator in the activation of the local inflammatory response during lung infections [28, 29]. In addition, increased concentrations of TREM-1 in the alveolar compartment have been found in patients with pneumonia and acute lung injury [30–32]. The expression of TREM-1 is upregulated on lung alveolar macrophages upon stimulation by bacterial pathogens, but expression levels are relatively low in patients with noninfectious inflammatory lung disease [29, 33, 34]. Recent evidence suggested that the level of TREM-1 in bronchoalveolar lavage fluid is a potential marker for pneumonia [31, 35]. Given the role that TREM-1 plays in the lung inflammatory response, genetic factors that affect TREM-1 secretion or function could be of important diagnostic and therapeutic relevance. This study was undertaken to determine if the TREM-1 SNP (A→T) SNP is associated with the development of pneumonia in burn-injured patients.

## 2. Materials and Methods

### 2.1. Study Design

We prospectively enrolled all patients admitted to the Burn Unit at Parkland Memorial Hospital (Dallas, TX, USA) with inhalation injury or with ≥10% total body surface area (TBSA) burns under a protocol approved by the Institutional Review Board at the University of Texas Southwestern Medical Center.

We excluded patients with significant nonburn-related trauma (Injury Score Scale (ISS) ≥ 16), traumatic or anoxic brain injury, spinal cord injury, known immunosuppression, chronic obstructive pulmonary disease, and malignancy, as well as those who failed to survive more than 48 hours after admission or who received palliative care only.

### 2.2. Clinical and Data Collection

Demographic data, a detailed medical history, clinical parameters, and laboratory data, together with information on the burn size and injury characteristics, were collected and recorded for all patients. 

### 2.3. Clinical Criteria for Ventilator-Associated Pneumonia

Diagnoses of pneumonia and VAP were based on the following criteria: clinical suspicion of pneumonia, as defined by the standard Center for Disease Control and Prevention criteria (CDC); the presence of new or progressive pulmonary infiltrates, together with at least two of the following: fever or hypothermia (temperature ≥ 38°C or ≤36°C); leukocytosis or leukopenia (≥12 10^9^/L or ≤3.5 10^9^/L); purulent respiratory secretions. In addition, a clinical pulmonary infectious score (CPIS) was calculated for all patients [36]. Bronchoalveolar lavage (BAL) with ≥10^4^ colony-forming units/mL was used as a confirmatory diagnostic modality for a positive BAL. Ventilator-associated pneumonia was defined by acquisition of the disease after ≥48 hours of mechanical ventilation.

Absence of pneumonia was established when another cause for pulmonary infiltrates was documented, and there was no bacterial growth in the BAL culture, in association with a full recovery without antimicrobial therapy. ACCP/SCCM guidelines were used to define sepsis, severe sepsis, and septic shock [37]. For this study, complicated sepsis was defined as all patients with severe sepsis and septic shock.

### 2.4. Blood Collection

Whole blood was collected in ethylene diamine tetraacetic acid (EDTA) tubes for DNA extraction from all patients enrolled in the study. Plasma was removed and buffy coats were stored at −4°C awaiting DNA extraction.

### 2.5. DNA Isolation and Genotyping

Genomic DNA was isolated by standard protocols as previously described [38]. Fragment containing the SNP was amplified from genomic DNA by polymerase chain reaction (PCR) using *Taq* DNA polymerase (Roche Diagnostics; Indianapolis, IN, USA). Thermal profile, reaction conditions, and primer sequence were optimized for the TREM-1 SNP (rs2234237) using the following base pair sequence: AGAACTCCGAGCTGCAACTAAATTA[**A**/**T**]CTGAGGAAAAGTATGAACTGAAAG. 

Amplification was carried out in a PTC 200 thermal cycler (MJ Research; Watertown, MA, USA). All of the genotypes were determined by Pyrosequencing [39] (Pyrosequencing AB, Westborough, MA) or TaqMan assays (Applied Biosystems, Inc., Foster City, CA, USA). 

### 2.6. Assay for LPS-Stimulated Human Whole Blood

Whole blood from seventy healthy volunteers was collected into EDTA Vacutainer tubes. Assays were performed after diluting the blood samples 9 : 1 in RPMI-1640 media supplemented with glutamine and antibiotics. Aliquots of 2 mL of diluted blood per well were equally divided into LPS stimulated (100 ng/mL of *E. coli* LCD25; List Biological Laboratories, Campbell, CA, USA) and non-LPS groups in 24-well plates (Corning Inc., Corning, NY, USA). Each experimental condition was conducted in triplicate. The supernatants were frozen at −80°C, and the cells were processed sTREM-1 analysis.

### 2.7. Soluble TREM-1 Measurements

Concentrations of soluble TREM-1 (sTREM-1) were measured in triplicates by an enzyme immunoassay (R&D Inc., Minneapolis, MN, USA) according to manufacturer instructions. The lower detection limit was 15.1 pg/mL. 

### 2.8. Risk Factors for Pneumonia

We included the presence of the TREM-1 SNP (A→T), as well as other known risk factors, including age, gender, race, use of cardiopulmonary resuscitation, presence of coma, preexisting conditions, percent of TBSA burned, percent of TBSA full thickness burn, type of burn, inhalation, duration of mechanical ventilation, length of stay (LOS), prior antimicrobial use, transfusions, and fluid requirements during resuscitation. All were included in the regression analysis. In addition, based upon previous studies of burn victims in our institution [40], patients were categorized according to the presence or absence of the following threshold values: age >50 years, full-thickness burn size ≥30% (FT), total burn surface area (TBSA), and inhalation injury; these variables were also evaluated as risk factors for pneumonia.

### 2.9. Statistical Analysis

Descriptive statistics included number (percentage) of categorical variables and median (range) for continuous data. Categorical data were compared using chi-square or Fisher's exact tests. Continuous data were analyzed by the Mann-Whitney-*U* test. We used multivariate logistic regression analysis to simultaneously evaluate the effects of multiple variables as risk factors for pneumonia. Adjusted odds ratios (aOR) are presented with their associated 95% confidence intervals (95% CI). We used SPSS 16 (Chicago, IL, USA) for statistical analyses. 

## 3. Results

### 3.1. Healthy Volunteers

**Figure 1:**
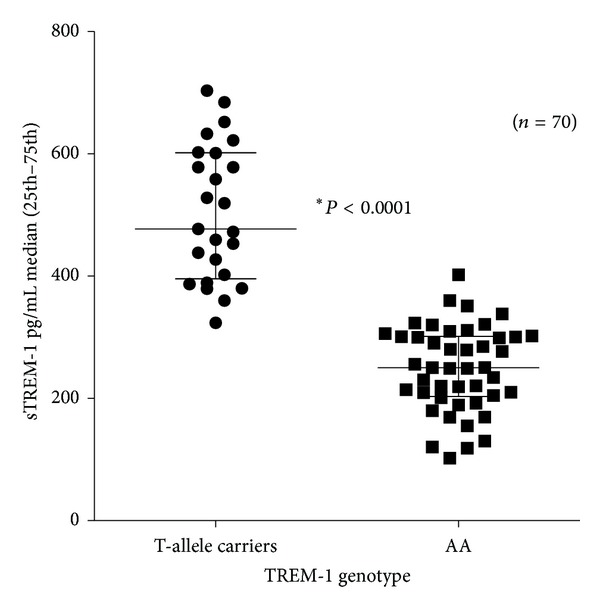
Soluble TREM-1 levels were significantly elevated in T allele carriers.

In examining the functionality of the TREM-1 polymorphism in our laboratory human whole blood from 70 healthy volunteers was stimulated with lipopolysaccharide (LPS). When stratified on TREM-1 genotype, carriers of the less common T allele had significantly higher TREM-1 levels (Figure 1).

### 3.2. Burn Patients

From March 2003 to March 2010, six hundred and ten patients were prospectively evaluated. Of those, five hundred and forty met the inclusion criteria. Baseline demographic data and genotype distribution are presented in Table 1. This cohort was composed of 540 relatively young (mean age = 38 years) and predominantly male patients (74%), with approximately 27% sustaining inhalation injury. The most common mechanism of burn was flame injury (68%). The most common preexisting conditions were alcohol abuse (13%), hypertension (11%), cardiovascular (8%), and diabetes mellitus (5%). Preexisting lung, liver, and renal diseases were uncommon (<3%). The most frequent sites of infection were lung (42%), wound (32%), urinary tract (17%), blood (15%), and line (7%). Pulmonary complications were the most common with pneumonia and ARDS developing in 209 (39%) and 121 (22%) members of the cohort, respectively. The majority of patients were intubated after admission to the hospital (92%). 

Two hundred and forty nine (46%) patients developed sepsis. The most common identified source of sepsis was the lung with 28%. The overall mortality in our cohort was 14%.

### 3.3. Comparison of Demographics and Clinical Outcome in Patients with and without Ventilator-Associated Pneumonia

Table 2 shows the differences in clinical and demographic variables between patients when stratified on development of VAP (VAP versus non-VAP). Although patients in the VAP group had significantly longer lengths of stay, higher rates of complicated sepsis, ARDS, and inhalation injury relative to non-VAP patients, there was no difference in mortality between the two groups.

### 3.4. Comparison of Demographics and Clinical Outcome among Genotypes

There were no clinical or demographic differences between patients with different TREM-1 genotypes. However, carriage of the T allele was associated with a significantly increased frequency of VAP when compared with AA homozygotes. The incidence of complicated sepsis was similar in both groups (Table 3).

### 3.5. Risk Factors for Pneumonia

In our unadjusted analysis, inhalation injury (OR 2.4, 95% CI 1.5–3.7, *P* = 0.02) *P* = 0, full-thickness burn size ≥ 30% TBSA (OR 2.6, 95% CI 1.7–4, *P* = 0.003) and carriage of the T allele (OR 6.3, 95% CI 4–9.3, *P* = 0.000) were significant clinical risk factors for VAP. Carriers of the T allele had a significantly higher risk of VAP. A significantly higher frequency of pneumonia (72%) was observed among patients who carried the T allele relative to the AA-homozygotes (29%) (*P* < 0.001). Importantly, the risk of pneumonia increased in a dose-dependent manner with the number of T alleles carried. VAP was identified in all but one of the eleven TT homozygous patients. 

A multivariate logistic regression analysis was performed with independent variables believed to influence the development of pneumonia. Associations with TREM-1 genotype were reanalyzed, adjusting for those variables found to be independently associated with outcome. After adjustment, full-thickness burn size ≥ 30%, inhalation injury, and TREM-1 T allele carriage remained significantly associated with risk factors for developing pneumonia (Table 4).

## 4. Discussion

In this study, we found a significant association between the nonsynonymous polymorphism in exon 2 of the TREM-1 gene (T allele) and pneumonia in mechanically ventilated burn patients. The TREM-1 T allele was an independent predictor of pneumonia. The frequency of the T allele correlated with pneumonia such, that only one case with the TT genotype failed to develop the phenotype of interest. To our knowledge this is the first time that this SNP has been evaluated for association with pneumonia.

A single nucleotide polymorphism (SNP) is defined as a mutation that occurs at a single, specific site in the DNA sequence. Many SNPs are located in the promoter or protein coding regions and directly affect protein abundance or function. Other SNPs may not appear to have a significant effect on abundance or function of a gene product, but could predispose individuals to disease or influence their response to injury [15, 16]. 

The TREM-1 candidate SNP was selected due to its nonsynonymous (missense) nature (Ser25Thr) and location within the second exon of the TREM-1 gene. By inducing a change in the amino acid sequence, the candidate SNP may influence the biologic function of TREM-1. Nonsynonymous coding SNPs, together with SNPs in regulatory regions, are believed to have the highest impact on phenotype, and they account for approximately half of the known gene variants responsible for human inherited disease [41]. More important, we demonstrated the functional effect of this SNP, on TREM-1 levels on LPS stimulated human whole blood; T allele carriage was associated with increased levels of soluble TREM-1.

TREM-1 is selectively expressed in the lungs of patients with pneumonia caused by extracellular bacteria but not in patients with tuberculosis [35]. Given the substantial evidence supporting the potential role of TREM-1 in the activation of the local inflammatory response during lung infections, any genetic variability affecting the production or function of TREM-1 following an infectious stimulus might have a significant impact upon the ensuing inflammatory response and subsequent clinical outcomes.

Genetic markers have the advantage of providing reliable information on susceptible individuals at risk of a condition and may guide therapeutic interventions. However, at the present time the clinical implications of these findings remain unclear, since there are likely additional genes and polymorphisms that may influence the development of pneumonia. Additionally, confirmation of the reported genetic association in other large and independent patient populations is warranted.

Nevertheless, these findings enhance our understanding of the pathobiology of lung infections and may aid in early identification of patients at increased risk for the development of pneumonia. Our current findings suggest that there may be a genetic predisposition to the development of pneumonia in patients with thermal injuries. 

## 5. Conclusions

To our knowledge, this is the first report describing the role of a TREM-1 genetic polymorphism in pneumonia susceptibility in humans. Carriers of the TREM-1 25 T allele appear to have a greater likelihood of developing ventilator-associated pneumonia. Early identification of these patients may lead to the development of strategies to reduce the risk of acquiring VAP in this high-risk group.

## Figures and Tables

**Table 1:** Demographics and clinical variables of 540 individuals who met enrollment criteria.

Variable	Total
Age, yr	38 (24–51)
Sex, male, *n* (%)	407 (74%)
Percent of TBSA burned (TBSA)	25 (16–41)
Percent of TBSA full thickness burn (FT)	11 (0–25)
LOS (length of stay)	21 (11–41)
LOS BICU (Burn Intensive Care Unit)	10 (3–24)
Apache II	12 (6–16)
Adult respiratory distress syndrome (ARDS)	119 (22%)
Ventilator-associated pneumonia (VAP)	209 (39%)
Inhalation injury	148 (27%)
Ventilator days	13 (6–23)
Sepsis	107 (20%)
Complicated sepsis (severe sepsis/septic shock)	143 (26%)
Mortality	74 (14%)
Race	
Asian	8 (2%)
Black	89 (16%)
Hispanic	126 (23%)
Caucasian	313 (57%)
Other	14 (2%)
TREM-1 genotype	
Homozygous **AA**	418 (77%)
Heterozygous **AT**	111 (21%)
Homozygous **TT**	11 (2%)

Continuous data are presented as medians (25th–75th percentiles). Categorical data are presented as number of patients (percentage).

**Table 2:** Differences in clinical variables between patients when stratified into groups by the presence of ventilator-associated pneumonia (VAP) or absence ventilator-associated pneumonia (NPNA).

Variable	VAP	NVAP	*P* value
(%) total burn surface area	32 (22–53)	30 (20–45)	0.08
(%) full thickness TBSA	17 (5–35)	6 (0–19)	<0.001
Inhalation	69 (37%)	78 (21%)	<0.001
Ventilator days	18 (11–30)	5 (2–10)	<0.001
Burn Intensive Care Unit Length of Stay	25 (15–44)	3 (0–11)	<0.001
Length of stay	40 (24–60)	14 (8–24)	<0.001
Age	37 (22–51)	38 (26–51)	0.19
Apache II	12 (9–17)	11 (6–18)	0.25
Adult respiratory distress syndrome (ARDS)	85 (41%)	34 (10%)	<0.001
Sepsis	158 (75%)	91 (27%)	<0.001
Complicated sepsis	86 (41%)	56 (17%)	<0.001
Mortality	30 (13%)	44 (14%)	0.72

**Table 3:** Stratification of demographic variables based upon TREM-1 genotype.

Variable	AA-homozygous (248)	T-allele carriers (78)	*P* value
Age, yr	38 (24–52)	37 (22–48)	0.35
Sex, male, *n* (%)	305 (73%)	95 (78%)	0.27
TBSA	30 (20–46)	30 (22–43)	0.90
Full thickness	15 (5–32)	12 (3–26)	0.35
Inhalation injury	110 (26%)	37 (30%)	0.38
ARDS	82 (19%)	37 (30%)	0.01
Apache II	11 (6–15)	9 (4–16)	0.54
Ventilator days	16 (7–24)	13 (8–24)	0.32
VAP	121 (29%)	88 (72%)	<0.001
Sepsis	189 (45%)	60 (49%)	0.44
Complicated sepsis	110 (26%)	32 (26%)	0.98
Mortality	60 (14%)	14 (11%)	0.41

**Table 4:** Significant risk factors for the development of VAP after burn injury following adjustment for multiple factors with multivariate logistic regression.

Risk factor	OR	95% CI	*P* value
Full-thickness burn ≥ 30% TBSA	1.8	1.2–2.8	0.003
TREM-1 T-allele carriage	6.3	4–6.3	<0.001
Inhalation injury	1.6	1–2.50	0.02
